# Frequency of Dietary Intake and Physical Activity in Older Adults: A Cross-Sectional Study

**DOI:** 10.3390/nu10121960

**Published:** 2018-12-11

**Authors:** Lovro Štefan, Lidija Petrinović, Goran Sporiš, Goran Vrgoč

**Affiliations:** 1 Faculty of Kinesiology, Horvaćanski zavoj 15, University of Zagreb,10 000 Zagreb, Croatia; lidija.petrinovic@kif.hr (L.P.); sporis.79@gmail.com (G.S.); 2 General Medical Hospital ‘Sveti Duh’, 10 000 Zagreb, Croatia; gvrgoc@gmail.com

**Keywords:** nutrition, elderly, exercise, public health, associations

## Abstract

Background: Evidence shows that diet is associated with physical activity. However, there has been a lack of studies addressing it in a population of older adults. Therefore, the main purpose of this study was to explore associations between various frequencies of dietary intake and physical activity in older adults. Methods: In this cross-sectional study, the participants were 810 older adults aged ≥85. Frequency of dietary intake and the level of physical activity were assessed using the Elderly Diet Index score and International Physical Activity questionnaire. Mutual associations were examined using generalized estimating equations with a logistic regression model. Results: ”Optimal” intake of fish and seafood (OR (odds ratio) = 1.40; 95% CI (95 percent confidence interval) 1.01 to 2.00), fruits (OR = 2.10; 95% CI 1.45 to 3.02), legumes (OR = 1.73; 95% CI 1.19 to 2.50), olive oil (OR = 1.83; 95% CI 1.09 to 3.08) and bread (OR = 4.62; 95% CI 3.05 to 6.99) and the total Elderly Diet Index score (OR = 4.99; 95% CI 3.20 to 7.70) were associated with ”sufficient” physical activity. When all dietary components were entered simultaneously into the model, ”optimal” intakes of meat (OR = 1.73; 95% CI 1.10 to 2.71), fish and seafood (OR = 2.26; 95% CI 1.46 to 3.51), cereals (OR = 1.75; 95% CI 1.02 to 3.25), fruits (OR = 1.52; 95% CI 1.02 to 2.26), legumes (OR = 1.48; 95% CI 1.10 to 1.93), and bread (OR = 5.14; 95% CI 3.24 to 8.15) were associated with ”sufficient” physical activity. Conclusions: Our study shows that the total Elderly Diet Index score is the strongest predictor associated with ”sufficient” physical activity in a population of older adults. Thus, policies aiming to improve overall diet in order to achieve higher levels of physical activity are warranted.

## 1. Introduction

Physical activity is an important determinant of health in older adults [1]. Maintaining good health represents a crucial factor for functional independence due to high nursing costs and premature disability [2]. Despite the health benefits of physical activity [3], older adults are still physically inactive [4,5]. According to the World Health Organization [6], older adults should do ”at least 150 min of moderate-intensity aerobic physical activity throughout the week or do at least 75 min of vigorous-intensity aerobic physical activity throughout the week or an equivalent combination of moderate- and vigorous-intensity activity.” Regular physical activity works beneficially against cardiovascular, metabolic, and mental diseases, as well as overall mortality [2]. However, only 9% of men and 6% of women aged ≥75 in Great Britain meet the recommended levels, and these numbers are declining [7]. 

A study by Pronk et al. [8] showed that among numerous lifestyle factors associated with developing chronic diseases (hypertension, heart disease, diabetes, depression, stroke, arthritis) and affecting health, physical activity and diet-quality practices (including the consumption of fat, fiber, fruit, and vegetables) represented the most significant predictors that could have an impact on such conditions. Additionally, these two factors often coexist, and changing one of them can potentially lead to changing the other [8]. According to the World Health Organization [6], both inadequate diet and physical inactivity are the main risk factors for developing non-communicable diseases, leading to poor health and death. Specifically, evidence shows that the multi-morbidity (having ≥2 chronic diseases) prevalence rates in older adults range between 55% and 98% [9], pointing out that strategies and policies targeting healthy behaviors are crucial. Recently, great attention has been given to exploring the associations between diet and physical activity. Studies among children and adolescents have shown that more active male individuals have higher intake of vegetables, meat, fish, eggs, and vegetarian products, while higher intake of bread and cereal products are found in females [10,11]. Similar associations have been found in the adult population, where more frequent consumption of fruit and vegetable juices have been associated with moderate- to vigorous-intensity physical activity [12,13]. In older adults, existing literature shows that they consume an inadequate amount of fruits and vegetables, and in general consume less nutrients which can prevent the development of chronic diseases [14]. A handful of existing studies have presented conflicting results. Most recently, del Mar Bibiloni et al. [15] conducted a study among Spanish older adults and showed no significant associations between the adherence to the Mediterranean diet and physical activity. Another two studies showed that higher adherence to the Mediterranean diet was associated with lower risk of low physical activity [16,17]. However, all three studies used the adherence to the Mediterranean diet as a proxy of dietary patterns. 

According to available literature, studies exploring the association between diet and physical activity in older adults are lacking. Since both behaviors are associated with each other and may prevent the development of chronic diseases and promote successful aging, it is necessary to explore whether a ”healthy” diet leads to recommended levels of physical activity. Therefore, the main purpose of the present study was to explore the association between various frequencies of dietary intake and physical activity in older adults.

## 2. Methods

### 2.1. Study Participants

Of 1040 individuals willing to participate, 810 (16.0% of men; mean age: 87.60 ± 2.44 years; mean height: 1.63 ± 0.09 m, mean weight: 71.60 ± 13.14 kg, mean body-mass index: 26.88 ± 4.34 kg/m^2^) met the inclusion criteria and were eligible for further analysis. More detailed information about the participants are described elsewhere [18]. Participants were selected from six conveniently selected neighborhoods in the city of Zagreb. After neighborhood selection, we spread information about the study using flyers and posters to inform future participants about the main aims and hypothesis. The inclusion criteria were: (1) age ≥ 85, (2) be free of cognitive diseases (the information about their cognitive functioning was confirmed through evidence provided by their physicians), and (3) be able to read and write individually. The testing protocol was conducted between August and September of 2018. All the procedures performed in this study were anonymous and in accordance with the Declaration of Helsinki. The Institutional Review Board of the Faculty of Kinesiology approved the study (Ethics code: 10/7/2018). Before the study, each participant gave their written informed consent to participate.

### 2.2. Physical Activity (Dependent Variable)

To assess physical activity in the last 7 days, we used the adapted version of the International Physical Activity Questionnaire - Short Form, a reliable and valid instrument designed to measure physical activity in respondents aged ≥65 [19]. The questionnaire provides information about the time and number of days spent in physical activity of light, moderate, and vigorous intensity. For each participant, we calculated the time spent in moderate and vigorous physical activity. The questionnaire has weak to moderate correlation with objectively measured physical activity (0.28–0.47), with observed systematic error. Its ability to identify low-active participants was 85%, yet its sensitivity to detect high-active participants was 81% [19]. According to the World Health Organization [6], those participants who participated in at least 150 min of moderate-intensity aerobic physical activity or at least 75 min of vigorous-intensity aerobic physical activity throughout the week were categorized as ”sufficiently” active, compared to those who did not meet the aforementioned criteria as ”insufficiently” active. 

### 2.3. Diet (Independent Variable)

To assess dietary patterns, we used the Elderly Dietary Index score [20]. It consists of ten dietary groups (i.e., meat, fish, fruits, vegetables, grains, legumes, olive oil, alcohol, bread, and dairy products). Scores from 1 to 4 are assigned to all components of the index. The Elderly Dietary Index total score ranges between 10 and 40, with higher values indicating healthier adherence to dietary recommendations for older adults. A study by Kourlaba et al. [20] showed that the Elderly Dietary Index score was associated with several health-related outcomes; that is, a higher score was associated with a lower likelihood of having at least one cardiovascular disease risk factor and obesity. More detailed information about the scoring protocol and dichotomization is presented in Table 1. 

### 2.4. Covariates

Height and weight of the participants were self-reported. We categorized the participants as normal (<25 kg/m^2^) compared to overweight/obese (≥25 kg/m^2^). Health was also self-reported and assessed by one question: ”How would you rate your health?“ with five possible answers: (1) very poor, (2) poor, (3) fair, (4) good, and (5) excellent. We categorized the outcome variable into ”good” (fair, good, and excellent) compared to ”poor” (poor and very poor) self-rated health perception. To assess sleep quality, we asked participants the following question: ”How would you rate your sleep quality?” with four possible answers: (1) very poor, (2) poor, (3) good, and (4) very good. We categorized the outcome as ”poor” (very poor and poor) compared to ”good” (good and very good) sleep quality. Psychological distress was assessed by using Kessler’s six-item questionnaire [21]. More detailed information about the scoring protocol is described elsewhere [21]. Socioeconomic status was categorized as (1) low vs. (2) middle/high. Whether or not participants had a chronic disease was collected by asking the question as follows: ”Have you ever been told by a doctor that you suffer from any kind of chronic disease?“ to be followed by a categorical yes/no response.

### 2.5. Data Analysis

Basic descriptive statistics of the study participants are presented as frequencies (*n*) and percentages (%). Differences between categorical variables were analyzed using the Chi-square test. To examine the associations between dietary patterns and physical activity, we calculated odds ratios (ORs) with 95% confident intervals (95% CIs) by using generalized estimating equations with Pearson’s deviance model to correct for potential neighborhood clustering. For the univariate model, we entered all ten components of the Elderly Diet Index separately into the model. For the multivariate model, we re-entered all ten components simultaneously into the model. For the univariate model, we additionally entered the total Elderly Diet Index score, but because of high multicollinearity with other components, we dropped it from the multivariate model. In both models, the ”outcome” variable was ”sufficient” physical activity and the ”referent” value was ”non-optimal” frequency of dietary intake. Both models were adjusted for body-mass index, self-rated health, sleep quality, socioeconomic status, psychological distress, and chronic diseases. The interaction effects between sex and physical activity (*p* = 0.344) and sex and diet (*p* = 0.278) were not statistically significant, so we dropped sex-stratified analyses. Significance was set up at α = 0.05. All the analyses were performed in the Statistical Package for Social Sciences Software, V.22 (Armonk, New York, United States of America; IBM corporation group).

## 3. Results

Basic descriptive statistics of the study participants are presented in Table 2. The majority of the sample had ”non-optimal” intakes of meat, fish and seafood, fruits, legumes, and olive oil. The higher percentage of participants reporting ”optimal” dietary intake of meat, fish and seafood, fruits, legumes, olive oil, dairy products, and bread were categorized as ”sufficiently” active. The Elderly Diet Index showed that the higher percentage of participants being in ”moderate-to-high” healthy diet were categorized as ”sufficiently” active (*p* < 0.001). Moreover, a higher percentage of participants with ”normal” body-mass index, ”good” self-rated health, ”good” sleep quality, and ”low” psychological distress were ”sufficiently” active.

Table 3 shows the associations between the frequency of dietary intake and physical activity. In the univariate model, “optimal” intake of fish and seafood (OR = 1.40; 95% CI 1.01 to 2.00), fruits (OR = 2.10; 95% CI 1.45 to 3.02), legumes (OR = 1.73; 95% CI 1.19 to 2.50), olive oil (OR = 1.83; 95% CI 1.09 to 3.08), and bread (OR = 4.62; 95% CI 3.05 to 6.99) and the total Elderly Diet Index score (OR = 4.99; 95% CI 3.20 to 7.70) were associated with “sufficient” physical activity. In the multivariate model, “optimal” intake of meat (OR = 1.73; 95% CI 1.10 to 2.71), fish and seafood (OR = 2.26; 95% CI 1.46 to 3.51), cereals (OR = 1.75; 95% CI 1.02 to 3.25), fruits (OR = 1.52; 95% CI 1.02 to 2.26), legumes (OR = 1.48; 95% CI 1.10 to 1.93), and bread (OR = 5.14; 95% CI 3.24 to 8.15) were associated with “sufficient” physical activity. Both models were adjusted for body-mass index, self-rated health, sleep quality, socioeconomic status, psychological distress, and chronic diseases.

## 4. Discussion

The main purpose of the present study was to explore the associations between various frequencies of dietary intake and physical activity in older adults. Specifically, our study showed that the total Elderly Diet Index score was the strongest positive predictor for higher levels of “sufficient” physical activity. In the multivariate model, we found that “optimal” consumption of meat, fish and seafood, cereals, fruits, legumes, and bread was associated with “sufficient” physical activity. It is possible that higher intake of meat, legumes, and bread were needed in order to restore empty energy deposits in elderly individuals who engaged in more physical activity. Our results are in line with previous studies [15,16,17,22]. Specifically, a study by del Mar Bibiloni et al. [15] showed that participants in the highest quartile of the Mediterranean dietary pattern were more likely to be classified in the second and third tertiles of leisure-time physical activity, and these associations remained after adjustment for body composition variables. The authors showed that participants classified in the fourth quartile of the Mediterranean dietary pattern were characterized by a high intake of whole-grain bread, rice, fish, fruits, vegetables, potatoes, legumes, nuts, olives and extra-virgin olive oil, honey, jam, and spices, and were more likely to practice leisure-time physical activity [15]. In the same study, the authors also found that the Mediterranean dietary pattern was associated with physical fitness, such as gait speed in men [15]. Another cross-sectional study showed that a healthy diet is inversely associated with frailty [22]. Similar findings have been observed in longitudinal studies [16]. Since physical fitness is one of the components of physical activity, our results might be interpreted alongside/in accordance with previous studies aiming to explore the associations between dietary patterns and physical fitness [15,23,24,25]. Specifically, high consumption of fruits, vegetables, whole wheat bread, and fatty fish was associated with higher handgrip strength [23]. Similarly, another study presented similar results, where seafood but no fish consumption was associated with lower body strength, agility, and endurance [15]. However, the aforementioned association between diet and physical fitness obtained by previous studies could only partially explain our results, since we used self-reported measures to assess physical activity in comparison to objectively assessed physical fitness in other studies. 

One previous study showed that the consumption of milk, yoghurt, and cheese was associated with higher levels of physical fitness and physical activity, which is contrary to our results [24]. Another study showed no association between dairy products and physical activity in a sample of older adults [15]. In the univariate model, we also observed that “optimal” consumption of meat and cereals was not significantly associated with “sufficient” physical activity. However, when all variables were entered simultaneously into the model, “optimal” consumption of meat and cereals became significantly associated with “sufficient” physical activity. Additionally, “optimal” olive oil consumption was significantly associated with “sufficient” physical activity, yet lost the effect in the multivariate model. Such findings could be explained by the fact that associations between the intakes of specific foods and nutrients and their interactive effects may be independent, and sometimes it is easier to detect the beneficial effects of a diet as a whole [25]. Such a statement could be confirmed by our findings, since the Elderly Diet Index (total score) was the strongest predictor of “sufficient” physical activity, compared to individual nutrients. 

Our study has several strong points. Firstly, we conducted a study among a relatively large sample of older adults (*n* = 810). Secondly, we adjusted for numerous socio-demographic (socioeconomic status) and health-related (body-mass index, self-rated health, sleep quality, psychological distress, chronic diseases) covariates.

However, our study also has some limitations. Firstly, due to a cross-sectional design, we could not determine the direction of the association. It is possible that “sufficient” physical activity led to better dietary intake. Secondly, we used self-reported measures, which might have led to measurement error. Our results should be interpreted with caution, since participants could have over/under reported their levels of physical activity and dietary intake. Additionally, one previous study showed that older adults do not provide accurate information about their height and weight, so the prevalence of overweight/obesity status must be interpreted with caution [26]. Finally, we did not include energy intake as a potential covariate, since it might have led to different associations. Thus, future studies using objective methods (e.g., accelerometers) and more detailed questionnaires (e.g., food frequency questionnaire) in a longitudinal design should be conducted in order to establish the causality of the association between diet and physical activity in a sample of older adults.

## 5. Conclusions

This study shows that more favorable dietary intake is associated with physical activity in a sample of older adults. That is, “optimal” intake of meat, fish and seafood, legumes, cereals, fruit, and bread is associated with more favorable levels of physical activity. Although we cannot make a conclusion about the causality of the association, given the importance of diet and physical activity in overall health, future strategies and policies aiming to enhance healthy dietary intake and increase levels of physical activity should be implemented within community settings.

## Figures and Tables

**Table 1:** Dichotomization of the frequency of dietary intake.

Frequency of Dietary Intake	Score Given to Each Category *	Dichotomized Score
**Meat**		
1–2 times/week	4	Optimal
<1 time/week	3	
Never/rarely	2	Non-optimal
≥3 times/week	1	
**Fish and Seafood**		
1–2 times/week	4	Optimal
≥3 times/week	3	
<1 time/week	2	Non-optimal
Never/rarely	1	
**Vegetables**		
Daily	4	Optimal
3–5 times/week	3	
1–2 times/week	2	Non-optimal
<1 time/week	1	
**Cereals**		
Daily	4	Optimal
3–5 times/week	3	
1–2 times/week	2	Non-optimal
<1 time/week	1	
**Fruits**		
Daily	4	Optimal
3–5 times/week	3	
1–2 times/week	2	Non-optimal
<1 time/week	1	
**Legumes**		
1–2 times/week	4	Optimal
<1 time/week	3	
Never/rarely	2	Non-optimal
≥3 times/week	1	
**Olive oil**		
Daily	4	Optimal
3–5 times/week	3	
1–2 times/week	2	Non-optimal
<1 time/week	1	
**Alcohol**		
0–2 glasses of wine/day	4	Optimal
No consumption	3	
3–4 glasses of wine/day	2	Non-optimal
>4 glasses of wine/day	1	
**Dairy**		
Nonfat milk/low-fat milk and low-fat cheese	4	Optimal
Nonfat milk and white or yellow cheese	3	
Full-fat milk and white or yellow cheese	2	Non-optimal
Low-fat milk and white or yellowcheese/full-fat milk and low-fatcheese	1	
**Bread**		
Whole-grain bread	4	Optimal
Combination of white and whole-grain bread	3	
White bread	2	Non-optimal
No consumption	1	
**Elderly Dietary Index (Total Score)**		
≥32 points	3	Moderate-to-high
29–31 points	2	
10–28 points	1	Low

*Higher score is associated with healthier dietary intake.

**Table 2:** Basic descriptive statistics of the study participants; Croatia (*n* = 810).

Study Variables	Total Sample (*n* = 810)	“Insufficient” Physical Activity (*n* = 630)	”Sufficient” Physical Activity (*n* = 180)	*p*-Value *
	*n* (%)	*n* (%)	*n* (%)	
**Meat**				
Non-optimal	587 (72.5)	461 (73.2)	126 (70.0)	
Optimal	223 (27.5)	169 (26.8)	54 (30.0)	0.396
**Fish and Seafood**				
Non-optimal	516 (63.7)	419 (66.5)	97 (53.9)	
Optimal	294 (36.3)	211 (33.5)	83 (46.1)	<0.001
**Vegetables**				
Non-optimal	214 (26.4)	164 (26.0)	50 (27.8)	
Optimal	596 (73.6)	466 (74.0)	130 (72.2)	0.633
**Cereals**				
Non-optimal	144 (17.8)	112 (17.8)	32 (17.8)	
Optimal	666 (82.2)	518 (82.2)	148 (82.2)	1.000
**Fruits**				
Non-optimal	541 (66.8)	445 (70.6)	96 (53.3)	
Optimal	269 (33.2)	185 (29.4)	84 (46.7)	<0.001
**Legumes**				
Non-optimal	458 (56.5)	371 (58.9)	87 (48.3)	
Optimal	352 (43.5)	259 (41.1)	93 (51.7)	0.013
**Olive oil**				
Non-optimal	718 (88.6)	564 (89.5)	154 (85.6)	
Optimal	92 (11.4)	66 (10.5)	26 (14.4)	0.144
**Alcohol**				
Non-optimal	26 (3.2)	21 (3.3)	5 (2.8)	
Optimal	784 (96.8)	609 (96.7)	175 (97.2)	0.815
**Dairy**				
Non-optimal	348 (43.0)	283 (44.9)	65 (36.1)	
Optimal	462 (57.0)	347 (55.1)	115 (63.9)	0.040
**Bread**				
Non-optimal	378 (46.7)	338 (53.7)	40 (22.2)	
Optimal	432 (53.3)	292 (46.3)	140 (77.8)	<0.001
**Elderly Diet Index Score**				
Low	666 (82.2)	554 (87.9)	112 (62.2)	
Moderate/high	144 (17.8)	76 (12.1)	68 (37.8)	<0.001
**Body-Mass Index**				
Overweight/obesity	512 (63.2)	410 (65.1)	102 (56.7)	
Normal	298 (36.8)	220 (34.9)	78 (43.3)	0.044
**Self-Rated Health**				
Poor	384 (47.4)	302 (47.9)	82 (45.6)	
Good	426 (52.6)	328 (52.1)	98 (54.4)	0.612
**Sleep Quality**				
Poor	302 (37.3)	254 (40.3)	48 (26.7)	
Good	508 (62.7)	376 (59.7)	132 (73.3)	<0.001
**Socioeconomic Status**				
Low	132 (16.3)	77 (12.2)	55 (30.6)	
Medium/high	678 (83.7)	553 (87.8)	125 (69.4)	<0.001
**Psychological Distress**				
High	200 (22.2)	176 (27.9)	24 (13.3)	
Low	610 (77.8)	454 (72.1)	156 (86.7)	<0.001
**Chronic Diseases**				
Yes	530 (65.4)	403 (64.0)	127 (70.6)	
No	280 (34.6)	227 (36.0)	53 (29.4)	0.110

*Chi-square test.

**Table 3:** Odds ratios for “sufficient” physical activity in the study participants; Croatia (*n* = 810).

Study Variables	Univariate Model *		Multivariate Model **	
	OR (95% CI)	*p*-Value	OR (95% CI)	*p*-Value
**Meat**				
Non-optimal	Ref.		Ref.	
Optimal	1.33 (0.90 to 1.96)	0.153	1.73 (1.10 to 2.71)	0.017
**Fish and seafood**				
Non-optimal	Ref.		Ref.	
Optimal	1.40 (1.01 to 2.00)	0.049	2.26 (1.46 to 3.51)	<0.001
**Vegetables**				
Non-optimal	Ref.		Ref.	
Optimal	0.98 (0.65 to 1.48)	0.938	0.81 (0.48 to 1.34)	0.405
**Cereals**				
Non-optimal	Ref.		Ref.	
Optimal	0.97 (0.60 to 1.56)	0.898	1.75 (1.02 to 3.25)	0.048
**Fruits**				
Non-optimal	Ref.		Ref.	
Optimal	2.10 (1.45 to 3.02)	<0.001	1.52 (1.02 to 2.26)	0.041
**Legumes**				
Non-optimal	Ref.		Ref.	
Optimal	1.73 (1.19 to 2.50)	0.004	1.48 (1.10 to 1.93)	0.035
**Olive oil**				
Non-optimal	Ref.		Ref.	
Optimal	1.83 (1.09 to 3.08)	0.023	1.13 (0.60 to 2.11)	0.701
**Alcohol**				
Non-optimal	Ref.		Ref.	
Optimal	0.95 (0.34 to 2.68)	0.922	1.25 (0.41 to 3.86)	0.692
**Dairy**				
Non-optimal	Ref.		Ref.	
Optimal	1.06 (0.73 to 1.55)	0.749	1.00 (0.65 to 1.54)	0.994
**Bread**				
Non-optimal	Ref.		Ref.	
Optimal	4.62 (3.05 to 6.99)	<0.001	5.14 (3.24 to 8.15)	<0.001
**Elderly Diet Index score**				
Low	Ref.		Ref.	
Moderate/high	4.99 (3.20 to 7.80)	<0.001		

* Examines separate associations between dietary habits and “sufficient” physical activity adjusted for body-mass index, self-rated health, sleep quality, socioeconomic status, psychological distress, and chronic diseases. ** Examines simultaneous associations between dietary habits and “sufficient” physical activity adjusted for body-mass index, self-rated health, sleep quality, socioeconomic status, psychological distress, and chronic diseases. *p* < 0.05.
